# Multi-fusion strategy network-guided cancer subtypes discovering based on multi-omics data

**DOI:** 10.3389/fgene.2024.1466825

**Published:** 2024-11-14

**Authors:** Jian Liu, Xinzheng Xue, Pengbo Wen, Qian Song, Jun Yao, Shuguang Ge

**Affiliations:** ^1^ School of Information and Control Engineering, China University of Mining and Technology, Xuzhou, China; ^2^ School of Medical Information and Engineering, Xuzhou Medical University, Xuzhou, China; ^3^ Department of Gynecology and Obstetrics, Taizhou Cancer Hospital, Wenling, China; ^4^ Department of Colorectal Surgery, Taizhou Cancer Hospital, Wenling, China

**Keywords:** cancer subtypes discovering, multi-omics data, clustering, deep learning, fusion strategy

## Abstract

**Introduction:**

The combination of next-generation sequencing technology and Cancer Genome Atlas (TCGA) data provides unprecedented opportunities for the discovery of cancer subtypes. Through comprehensive analysis and in-depth analysis of the genomic data of a large number of cancer patients, researchers can more accurately identify different cancer subtypes and reveal their molecular heterogeneity.

**Methods:**

In this paper, we propose the SMMSN (Self-supervised Multi-fusion Strategy Network) model for the discovery of cancer subtypes. SMMSN can not only fuse multi-level data representations of single omics data by Graph Convolutional Network (GCN) and Stacked Autoencoder Network (SAE), but also achieve the organic fusion of multi- -omics data through multiple fusion strategies. In response to the problem of lack label information in multi-omics data, SMMSN propose to use dual self-supervise method to cluster cancer subtypes from the integrated data.

**Results:**

We conducted experiments on three labeled and five unlabeled multi-omics datasets to distinguish potential cancer subtypes. Kaplan Meier survival curves and other results showed that SMMSN can obtain cancer subtypes with significant differences.

**Discussion:**

In the case analysis of Glioblastoma Multiforme (GBM) and Breast Invasive Carcinoma (BIC), we conducted survival time and age distribution analysis, drug response analysis, differential expression analysis, functional enrichment analysis on the predicted cancer subtypes. The research results showed that SMMSN can discover clinically meaningful cancer subtypes.

## 1 Introduction

Cancer is a heterogeneous disease characterized by diverse pathogenic mechanisms and clinical features (Wang et al., 2023). Research has shown that genomic alterations, such as copy number variations and somatic mutations, can lead to cancer development (Xu et al., 2023). Due to high heterogeneity, patients with similar phenotypes often exhibit different genomic changes, resulting in varied symptoms among cancer subtypes, which significantly impacts clinical diagnosis and prognosis (Jin et al., 2023). A major focus in current cancer research is predicting molecular subtypes using multi-omics data (Livesey et al., 2023; Chen et al., 2023). Classifying cancer subtypes can enhance our understanding of cancer pathogenesis and aid in personalized treatment approaches (Sosinsky et al., 2024).

Early research on cancer subtype discovery primarily concentrated on single omics data, such as gene expression data, using general clustering algorithms (Rappoport and Shamir, 2018). However, with the rapid accumulation of diverse omics data and the development of extensive cancer genome databases, the field has evolved significantly. One notable resource is The Cancer Genome Atlas (TCGA) (Akbani et al., 2014; Baird and Roychoudhuri, 2024), which has extensively studied multi-omics data from various cancer types across numerous patient samples. This wealth of sequencing data offers unprecedented opportunities to utilize multi-omics approaches for the discovery of cancer subtypes, paving the way for more precise and comprehensive cancer research and treatment strategies.

Researchers have proposed various methods for predicting cancer subtypes using multi-omics data. The simplest approach involves concatenating different biological data to form a single input matrix, followed by applying general clustering methods to identify cancer subtypes. For instance, Wu et al. (2015) introduced a comprehensive probability model called LRAcluster, based on low-rank approximation, to swiftly mine the shared main features across multiple omics data types. However, such methods often overlook differences in distribution and dimensionality among omics data, making it challenging to accurately characterize the input features. To address this, more sophisticated clustering strategies have been developed that consider the unique characteristics of each data source. The iCluster model (Shen et al., 2009) assumes that each omics dataset contains latent variables and employs a sparse method for gene selection and clustering. However, iCluster is limited to clustering continuous data types. Building on this, Mo et al. (2013) proposed iClusterPlus, an algorithm capable of jointly modeling multiple types of omics data, including continuous, count, and binary data. Additionally, Shi et al. designed the PFA algorithm (Shi et al., 2017), which maps each type of omics data to its corresponding low-dimensional space and performs automated information alignment and bias correction to achieve global pattern fusion in the feature space. These advancements offer more accurate and nuanced approaches to cancer subtype prediction, leveraging the full potential of multi-omics data.

The approaches mentioned primarily emphasize the representational characteristics of omics data while neglecting the structural insights that can illuminate similarities among patients, which are crucial for effective data learning. Spectral clustering (Luxburg, 2007) stands out as a method that captures such structural features by constructing graphs from data samples and leveraging graph-based clustering. Building on spectral clustering, various data integration algorithms have been developed. For instance, Wang et al. (2014) introduced the SNF method, which establishes similarity networks for diverse omics data types and integrates these networks using non-linear fusion techniques, thereby exploiting the complementary nature of the data. Expanding on these concepts, Ma and Zhang (2017) proposed the ANF method, which constructs K-nearest neighbor (KNN) networks for different omics datasets. These individual networks are then amalgamated into a unified fusion network using a random walk approach. To address the optimization challenges of spectral clustering, Yu et al. (2019) employed a linear search technique on the Stiefel manifold space, culminating in the MVCMO algorithm designed specifically for clustering multi-omics data. These advancements not only enhance our ability to extract meaningful insights from omics data but also underscore the importance of structural information for more robust data analysis and learning.

Deep learning has rapidly emerged as a research hotspot in the field of Artificial Intelligence (AI), especially in image data processing. Many deep learning-based methods for processing omics data have also been proposed to address the problem of cancer subtype discovery. Chen et al. (2020) proposed the DeepType algorithm for cancer classification, which combines supervised learning, unsupervised learning, and dimensionality reduction to learn data representations with clustering structures. Way and Greene (2018) utilized Variational Autoencoders (VAE) to compress gene expression features, thereby uncovering biologically relevant latent spaces. Xu et al. (2019) employed a Stacked Autoencoder (SAE) model to learn high-level representations of each omics data type, integrating these representations into an autoencoder layer to achieve a complex representation. They then used a Deep Flexible Neural Forest (DFNForest) model to classify the samples. These methods leverage deep learning to extract high-level feature representations from omics data and predict cancer subtypes based on these learned features. However, they often do not utilize the structural information inherent in omics data, which can be crucial for a more comprehensive understanding and prediction of cancer subtypes.

Graph Convolutional Networks (GCNs) (Thomas and Kipf, 2017) extend Convolutional Neural Networks (CNNs) to graph structures from the perspective of spectral theory (Bruna et al., 2013) (Defferrard et al., 2016). GCNs integrate the connectivity and characteristics of graph-structured data, and it has been demonstrated that GCNs and their variants (Hamilton et al., 2017; Veličković et al., 2017; Dai et al., 2018; Chen et al., 2017) significantly outperform Multi-Layer Perceptron (MLP) networks and traditional graph learning methods (Tang et al., 2015; Perozzi et al., 2014; Grover and Leskovec, 2016). To obtain high-level representations and fully utilize the spatial structure characteristics of omics data, we propose a new multi-omics deep clustering algorithm for discovering cancer subtypes, called Self-supervised Multi-fusion Strategy Network (SMMSN). SMMSN utilizes GCNs and SAEs to achieve the fusion of representation and structural information. It introduces various multi-omics data fusion strategies, ultimately achieving clustering through a self-supervised mechanism. This approach ensures efficient integration and utilization of information within and between omics data, leading to more accurate and insightful cancer subtype discovery.

The main contributions of our work are as follows.

(1) Integration of Structured and Representation Information. We introduce a novel method for integrating both structured and representation information within omics data. This approach aims to comprehensively harness and effectively learn the diverse and rich information inherent in multi-omics datasets.
(2) Multi-omics Data Fusion. We present two distinct methods for fusing multi-omics data: error reconstruction fusion and adaptive weighting network fusion. These methods are tailored to different aspects of data representation fusion, offering versatile strategies adapted to specific data characteristics.
(3) Dual Self-supervised Learning. We design a dual self-supervised learning module to perform unsupervised training on fused representations. By leveraging a self-supervised loss function, SMMSN enables the discovery of cancer subtypes from multi-omics fusion data without the need for real labels.
(4) Experimental Validation and Clinical Relevance. Experimental results compared with other algorithms and Kaplan-Meier survival curves demonstrated that SMMSN effectively distinguishes cancer subtypes with significant survival differences. In our analysis of Glioblastoma Multiforme (GBM) and Breast Invasive Carcinoma (BIC), the findings underscored SMMSN’s capability to discover clinically relevant cancer subtypes.

## 2 Materials and methods

**FIGURE 1:**
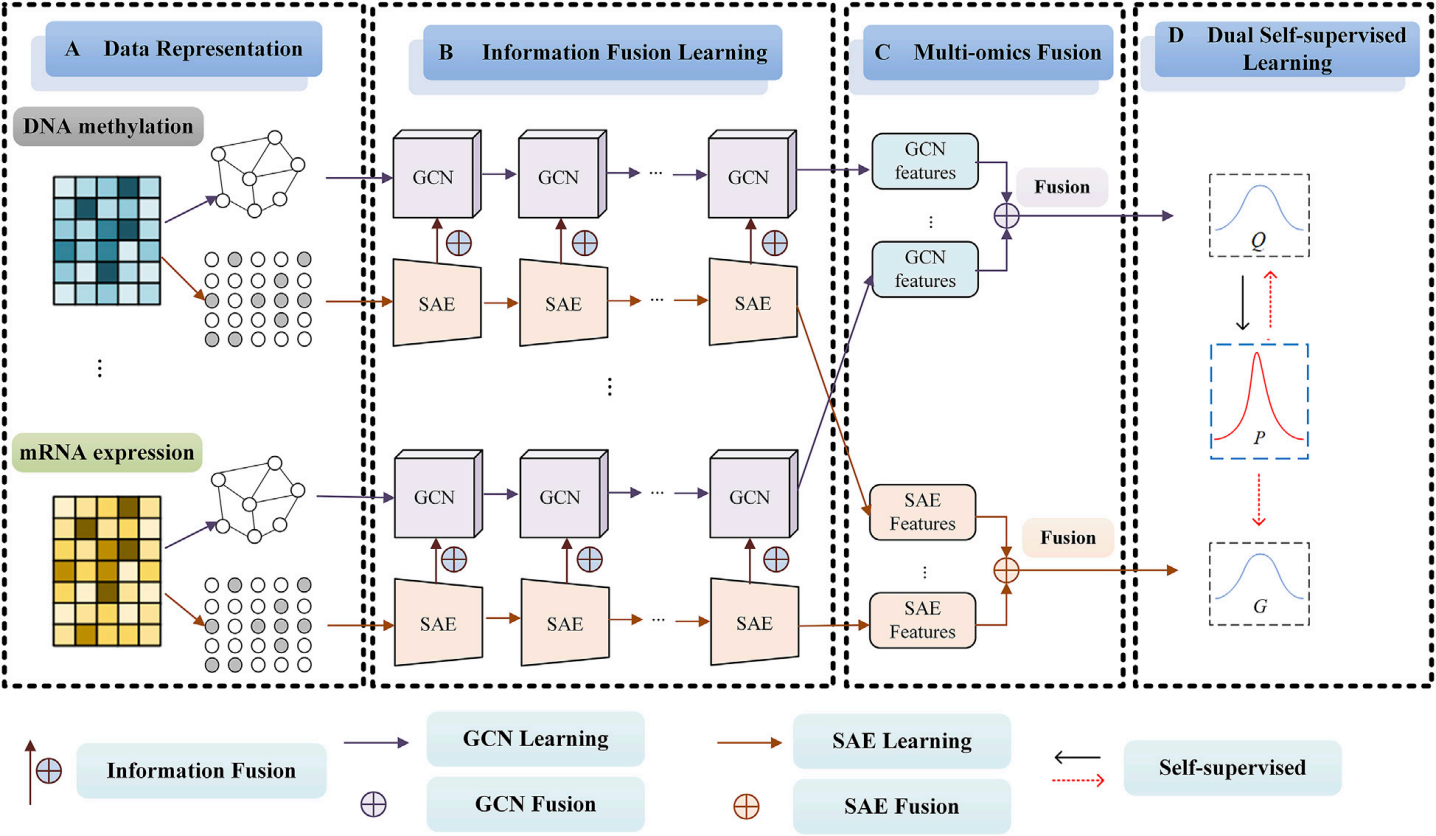
The framework of our proposed SMMSN model for cancer subtype discovery based on multi-omics (Take DNA methylation data and mRNA expression data, for example,). SMMSN contains four main modules: **(A)** Date Representation, **(B)** Representation Fusion Learning, **(C)** Multi-omics Fusion, **(D)** Dual Self-supervised Learning.

The framework of our SMMSN for cancer subtype discovery based on multi-omics (Take DNA methylation data and mRNA expression data, for example,) is shown in Figure 1. SMMSN contains four main modules: A) Date Representation, B) Information Fusion Learning, C) Multi-omics Fusion, D) Dual Self-supervised Learning. The general clustering process of SMMSN is presented as follows.

**① Date Representation Module**. For the v -th omics data $X_{v}$ , a KNN graph $A_{v}$ is constructed to obtain the structure information. At the same time, the feature representation is initialized and taken as input to the SAE network.
**② Information Fusion Learning Module**. Based on the KNN graph $A_{v}$ , a multi-layer GCN model is used to obtain the high-order structure representation $G_{v}^{l-1}$ , which is the output of the l−1 layer in the neural network. At the same time, SAE is used to learn the feature representation $Z_{v}^{l-1}$ of the omics data by using $X_{v}$ . Then $G_{v}^{l-1}$ and $Z_{v}^{l-1}$ are combined to obtain a joint representation $H_{v}^{l-1}$ that contains both high-level structural information and feature information. The output of the SAE is $Z_{v}^{l}$ , and the output of the GCN is $G_{v}^{l}$ which is obtained by $H_{v}^{l-1}$ . In this way, the structure information and feature information can be introduced into the deep clustering model through $G_{v}^{l}$ .
**③ Multi-omics Fusion Module**. According to the characteristics of different data representations, two data fusion methods are proposed to integrate the information of multiple omics data. For the GCN network output $G_{v}^{l}$ , an adaptive weighting network is designed to obtain GCN fusion representation $G_{fusion}$ . For the SAE network output $Z_{v}^{l}$ , an error reconstruction method is proposed to obtain SAE fusion representation $Z_{fusion}$ .
**④ Dual Self-supervised Learning Module**. A dual self-supervised module is used to jointly learn $G_{fusion}$ and $Z_{fusion}$ to achieve end-to-end training of the entire model. Firstly, the probability distribution matrix Q containing the sample clustering information is calculated according to $Z_{fusion}$ . Through learning high-confidence distribution to make the data representation closer to the cluster center and the target probability distribution matrix P is obtained. We use the softmax function to perform multi-classification on $G_{fusion}$ , and obtain the probability distribution matrix G . Finally, P is used to perform supervised training on the probability distribution matrices Q and G .

After the iteration is completed, the probability distribution matrix G contains both the feature representation information and structure information of the data. Therefore, the cluster label Y is calculated according to G .

### 2.1 Data representation module

Given multiple omics datasets $X=\left\{X_{1},X_{2},\cdots ,X_{v},\cdots ,X_{V}\right\}$ , where V represents the number of datasets, $X_{v}\in R^{N\times m_{v}}$ is the v -th omics data in X , $m_{v}$ represents that the v -th omics data has m genes (features), and N represents the number of patients (samples). Prior to implementing our SMMSN model, we carried out several preprocessing steps to address outliers within the multi-omics data. First, any patient with more than 20% missing information in a particular data type was excluded from analysis. Similarly, biological features (such as mRNA expression) with over 20% missing values across all patients were also removed. Additionally, normalization was applied using the following formula:

$$f_{n}=\frac{f-E\left(f\right)}{\sqrt{\text{Var}\left(f\right)}}$$ (1)

In Equation 1 f is any biological feature, $f_{n}$ is the corresponding feature after normalization, $E\left(f\right)$ and $\text{Var}\left(f\right)$ represent the mean and variance of f , respectively.

The aim of data representation module is to construct the input of GCNs and SAEs. For GCNs, we use the adjacency matrices constructed from the original data matrices of different omics as input. Since the adjacency matrix represents the relationship information between patient samples, and the number of patients is consistent across all omics data, the input matrix for each omics data in the GCN is of size N×N *,* where *N* is the number of patients. For SAEs, the input feature dimensions of different omics data can vary, but after being compressed by the encoder, the encoded representations of each type of omics data can be mapped to the same latent space dimension. This means that although the input features of the omics data differ, their output feature dimensions can be aligned through the encoder. In this way, even if the original feature dimensions of different omics data are inconsistent, the autoencoder can compress them into feature representations of the same dimension, allowing these features to be processed consistently in subsequent fusion operations.

Therefore, we take the matrix after the initialization of the omics data as the SAE input, and the *v*th omics data is still represented by $X_{v}$ . A KNN graph is constructed as the input of GCN based on each omics data. For each sample of each omics data, we select its top-*K* similar samples as neighbors to calculate the similarity between it and each neighbor, and then construct the similarity matrix $S_{v}\in R^{N\times N}$ . We use the heat kernel method to construct the KNN graph, and the similarity between the two samples i and j can be written as

$$S_{v}^{ij}=e^{-\frac{{\left\|X_{v}^{i}-X_{v}^{j}\right\|}^{2}}{\sigma }}$$ (2)

In Equation 2 σ represents heat kernel parameter. Then the top-*K* similar samples of each omics data are defined as neighbors to form the adjacency matrix $A_{v}\in R^{N\times N}$ .

### 2.2 Information fusion learning module

**FIGURE 2:**
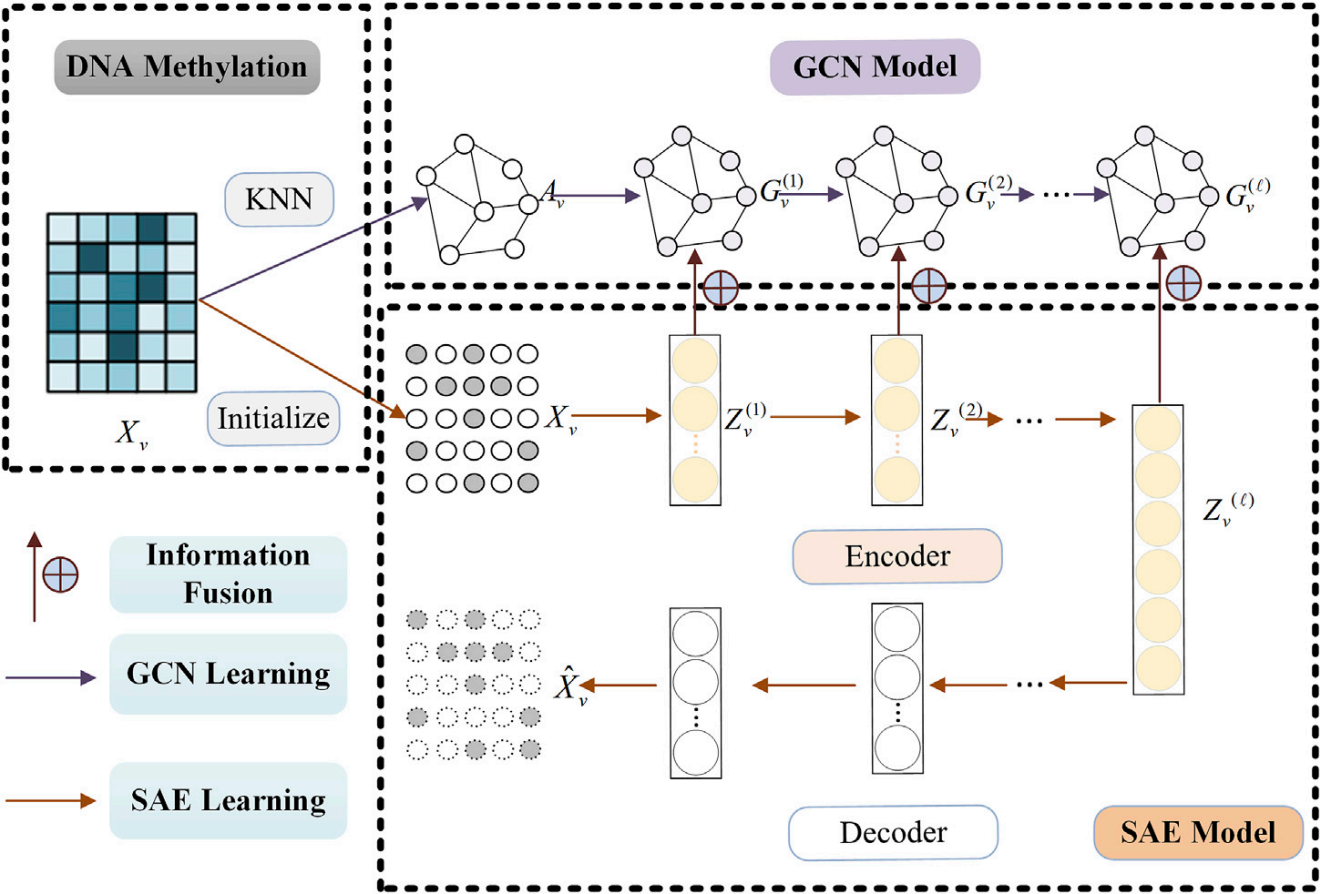
Information fusion learning process of single omics data (Take DNA Methylation data, for example,) by combining SAE and GCN model.

This subsection contains three processes: GCN learning, SAE learning and information fusion learning. The whole information fusion learning process of single omics data can be found in Figure 2 (Take DNA Methylation data for example).

#### 2.2.1 Stacked autoencoder learning

It is critical to learn effective feature representation in clustering tasks. Compared with traditional methods, deep learning methods can extract more advanced data feature representations and are widely used in various fields. In order to extract the high-level feature representation of omics data, we use the Stacked Autoencoder (SAE) model with the strongest generalization performance to learn the original omics data. The training process of SAE model can be found in Figure 2 (See SAE Model).

Suppose there are l layers in the SAE. In the encoder stage, when SAE is used to learn omics data $X_{v}$ , the learning of the l -th layer is written as $Z_{v}^{\left(l\right)}$

Zvl=ϕWevlZvl−1+bvle (3)

In Equation 3 ϕ is the activation function of the full connection layer. Here we use the LeakyRELU activation function. Wvle and bvle are the weight matrix and bias of the l -th layer in the encoder, respectively. When the encoder starts learning, the feature representation is initialized as: $Z_{v}^{\left(0\right)}=X_{v}$ .

In the decoder stage, the input data is reconstructed through multiple fully connected layers, which can be written as

Zvl=ϕWvldZvl−1+bvld (4)

In Equation 4 Wvld and bvld are the parameters of l -th layer in the decoder.

The final output $Z_{v}^{\left(l\right)}$ is the output ${\hat{X}}_{v}$ of SAE: ${\hat{X}}_{v}=Z_{v}^{\left(l\right)}$ . We hope that ${\hat{X}}_{v}$ can reconstruct the original omics data $X_{v}$ as much as possible, and then use the following loss function in Equation 5 for SAE model training

$$L_{res}=\frac{1}{2N}\sum_{v}^{V}{\left\|{\hat{X}}_{v}-X_{v}\right\|}_{F}^{2}$$ (5)

#### 2.2.2 Graph convolutional network learning

SAE can learn the advanced feature representation of omics data, but it does not consider the structural information among omics data samples. We introduce Graph Convolutional Network (GCN) to learn the structural representation of each omics data. The training process of GCN model can be found in Figure 2 (See GCN Model).

For omics data $X_{v}$ , GCN learns the structural representation $G_{v}^{\left(l\right)}$ of the l -th layer through the following convolution operations

$$G_{v}^{\left(l\right)}=C\left({\hat{A}}_{v}G_{v}^{\left(l-1\right)}W_{v}^{\left(l-1\right)}\right)$$ (6)

where $W_{v}^{\left(l-1\right)}$ is the weight matrix of l−1 -th layer. ${\hat{A}}_{v}=A_{v}+I$ , where I is an identity matrix. According to Equation 6, GCN can learn the representation $G_{v}^{\left(l\right)}$ of the next layer through $G_{v}^{\left(l-1\right)}$ , $W_{v}^{\left(l-1\right)}$ and the adjacency matrix ${\hat{A}}_{v}$ .

#### 2.2.3 Information fusion learning

The information fusion learning process of single omics data by combining SAE and GCN model can be found in Figure 2. Considering both $Z_{v}^{\left(l-1\right)}$ and $G_{v}^{\left(l-1\right)}$ , we can obtain a joint representation $H_{v}^{\left(l-1\right)}$ with more effective information through the following formula

$$H_{v}^{\left(l-1\right)}=\left(1-\varepsilon \right)G_{v}^{\left(l-1\right)}+\varepsilon Z_{v}^{\left(l-1\right)}$$ (7)

where ε is the balance parameter used to balance the relationship between the two representations $Z_{v}^{\left(l-1\right)}$ and $G_{v}^{\left(l-1\right)}$ . For simplicity, we set it to 0.5. Through Equation 7, we have realized the connection between SAE and GCN network. And $H_{v}^{\left(l-1\right)}$ contains both feature representation information and structure representation information.

Next, we need to learn the l -th layer representation $G_{v}^{\left(l\right)}$ of GCN. At this time, $H_{v}^{\left(l-1\right)}$ is taken as the input of GCN. Then we have

$$G_{v}^{\left(l\right)}=C\left({\hat{A}}_{v}H_{v}^{\left(l-1\right)}W_{v}^{\left(l-1\right)}\right)$$ (8)

In the traditional GCN model, after the multi-layer graph convolution operation is adopted, the characteristics of different nodes tend to be homogenized, that is, the characteristics of all nodes within the same connected component are almost the same. This is the so-called over-smoothing phenomenon. The representation information learned by the SAE in each layer is very different, and in Equation 8, the joint representation $H_{v}^{\left(l-1\right)}$ contains both the feature information learned and the structured information learned. Therefore, the existence of Equation 7 can alleviate the over-smoothing problem of GCN.

It is worth noting that the input data matrix $G_{v}^{\left(1\right)}$ of the first layer can be calculated by using omics data $X_{v}$ . $G_{v}^{\left(1\right)}$ can be defined by Equation 9

$$G_{v}^{\left(1\right)}=C\left({\hat{A}}_{v}X_{v}W_{v}^{\left(1\right)}\right)$$ (9)

The final output of GCN is determined according to Equation 10

$$G_{v}^{\left(l\right)}=C\left({\hat{A}}_{v}H_{v}^{\left(l-1\right)}W_{v}^{\left(l-1\right)}\right)$$ (10)

### 2.3 Multi-omics fusion module

**FIGURE 3:**
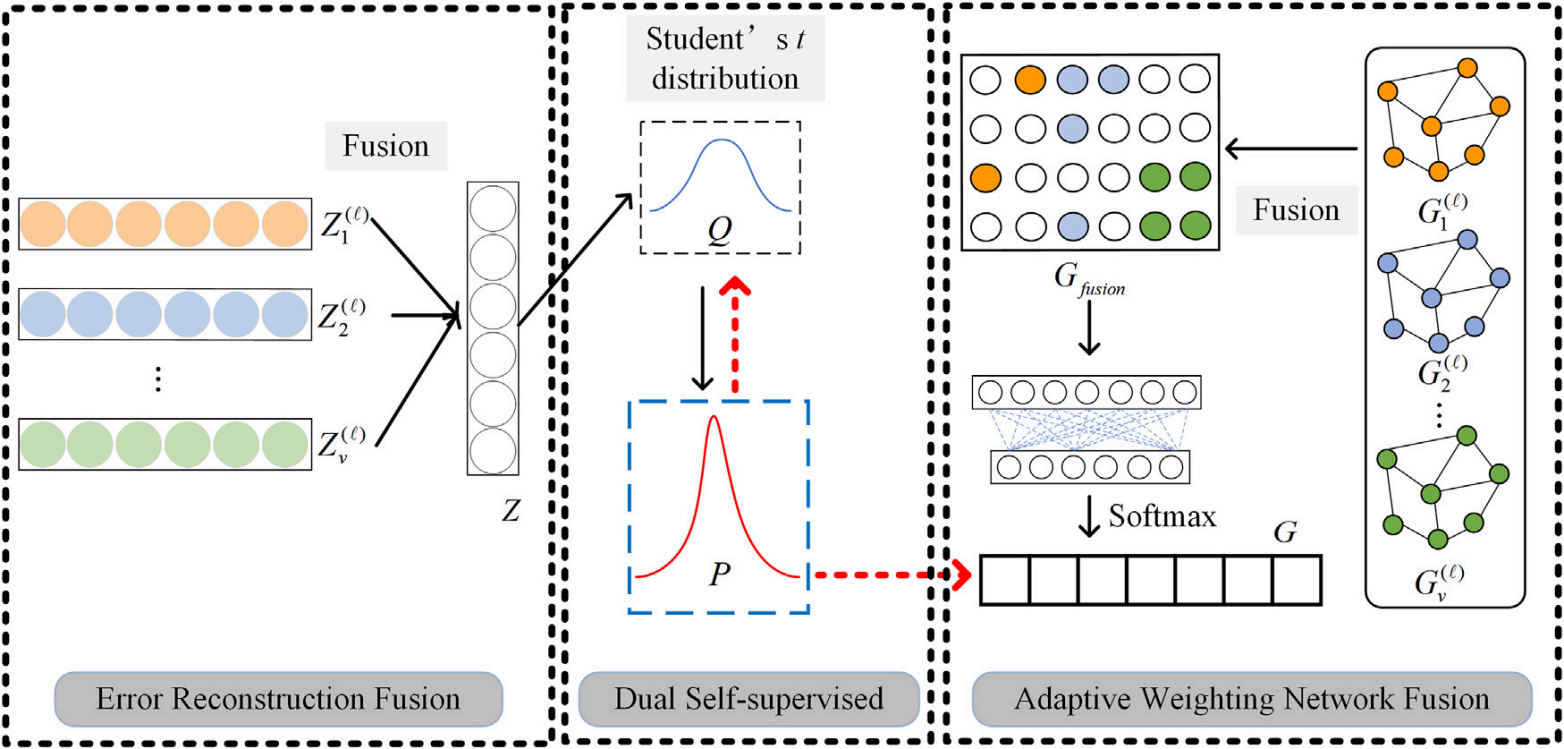
Graphical illustration of two multi-omics data fusion strategies and dual self-supervised learning.

After learning the feature representation and structural representation of any kind of omics data, in order to realize the further clustering task, it is necessary to fuse multi-omics data representations. Based on the different characteristics of omics data representations, we propose two multi-omics data fusion ideas: adaptive weighting network fusion and error reconstruction fusion, to implement Feature Representation Fusion (FRF) and Structural Information Fusion (SIF), respectively. The detailed fusion process can be found in Figure 3.

For the GCN output $G_{v}^{\left(l\right)}$ of each omics data, we connect them in series and propose an adaptive weighting network for fusion to obtain a fusion representation $G_{fusion}\in R^{N\times N}$

$$G_{fusion}=\left[G_{1}^{\left(l\right)}∥G_{2}^{\left(l\right)}∥\cdots ∥G_{V}^{\left(l\right)}\right]W_{fusion}$$ (11)

where $W_{fusion}\in R^{VN\times N}$ is a weight matrix that needs to be learned in the fusion process. Since $G_{v}^{\left(l\right)}$ contains the structural information of the omics data, it is necessary to consider the correlation between the samples in the fusion process. Therefore, in Equation 11, we first connect $G_{v}^{\left(l\right)}$ of each omics data to form an overall joint matrix, and then use $W_{fusion}$ to perform weighted learning, so that the adaptive weighting of all samples of all omics data is realized. After obtaining $G_{fusion}$ , we use the softmax function to perform multiple classifications to obtain a probability distribution matrix G , where $g_{ij}\in G$ denotes the probability that the sample i belongs to category j .

For the SAE output $Z_{v}^{\left(l\right)}$ of each omics data, we propose an error reconstruction fusion method to obtain a fusion representation $Z\in R^{N\times N}$ . First, $Z_{v}^{\left(l\right)}$ is initialized, and then it is learned according to the following loss function

$$L_{fus}=\sum_{v=1}^{V}{\left\|Z-Z_{v}^{\left(l\right)}\right\|}_{F}^{2}$$ (12)

Following Equation 12, our method can learn the fusion representation with the smallest error of all omics data feature representation through this data reconstruction idea.

### 2.4 Dual self-supervised learning module

Traditional SAE and GCN are unsupervised learning and semi-supervised learning algorithms respectively, which cannot be directly applied to clustering problems. In this paper, the dual self-supervised method is used to uniformly train the multi-omics data fusion representation learned by SAE and GCN to realize the clustering task. Graphical illustration of dual self-supervised learning is given in Figure 3.

Firstly, K-means algorithm is adopted to cluster the fusion representation Z of SAE, and get c initial cluster centers, where c is the number of clusters. For the i -th sample $Z_{i}$ (the i -th row of Z ) and the j -th cluster center $\mu_{j}$ of Z , we use the student’s *t* distribution in Equation 13 (Dunnett and Sobel, 1954) to measure the similarity between them (Tao et al., 2019; Wang et al., 2018)

$$q_{ij}=\frac{{\left(1+{\left\|Z_{i}-\mu_{j}\right\|}^{2}/\delta \right)}^{-\frac{\delta +1}{2}}}{\sum_{j^{\prime }}{\left(1+{\left\|Z_{i}-\mu_{j^{\prime }}\right\|}^{2}/\delta \right)}^{-\frac{\delta +1}{2}}}$$ (13)

where δ is the degree of freedom of student’s *t* distribution, $q_{ij}$ is the probability that the i -th sample is allocated to the j -th cluster center. The probability distribution matrix of all sample assignments can be denoted as Q , and $q_{ij}\in Q$ .

Then we optimize Z by learning high-confidence assignments to make the data representation closer to the cluster center. In Equation 14, the target distribution matrix $p_{ij}\in P$ can be obtained according to Q

$$p_{ij}=\frac{q_{ij}^{2}/f_{j}}{\sum_{j^{\prime }}q_{ij}^{2}/f_{j^{\prime }}}$$ (14)

where $f_{j}=\sum_{i}q_{ij}$ . In P , all assignments have higher confidence.

In order to minimize the loss between Q and P , KL divergence is used as the loss function

$$L_{clu}=\text{KL}\left(P∥Q\right)=\sum_{i}\sum_{j}p_{ij}\log\frac{p_{ij}}{q_{ij}}$$ (15)

Equation 15 can make the data representation closer to the cluster center, which is conducive to data clustering. P is calculated by Q , and the update of Q needs to rely on P . Therefore, this is a self-supervised learning mechanism.

We also perform self-supervised learning on the fusion representation of GCN. Since we have obtained the probability distribution matrix G of GCN output, we can directly use P and G to perform supervised learning. That is

$$L_{gcn}=\text{KL}\left(P∥G\right)=\sum_{i}\sum_{j}p_{ij}\log\frac{p_{ij}}{g_{ij}}$$ (16)

Through the above-mentioned dual self-supervised learning mechanism, the target distribution P conducts supervised learning on Q and G respectively in Equations 15, 16, so that the fusion output representations of GCN and SAE are unified under the same optimization framework. After iteration and update, the final training results tend to be consistent.

In conclusion, the overall loss function of the proposed SMMSN framework is defined as Equation 17

$$L=L_{res}+\lambda_{1}L_{fus}+\lambda_{2}L_{clu}+\lambda_{3}L_{gcn}$$ (17)

where $\lambda_{1}$ , $\lambda_{2}$ and $\lambda_{3}$ are hyperparameters used to balance different loss functions.

Since the final output G of SMMSN model contains both the representation information and structure information of the data, in Equation 18, we use G to achieve clustering. Then the label $y_{i}\in Y$ of sample i can be calculated by the following formula

$$y_{i}=\arg\;\max_{j}\;g_{ij}$$ (18)

where $g_{ij}\in G$ .

## 3 Results and discussion

In the experimental phase, we validated the effectiveness of our proposed algorithm using two major categories of real-world cancer multi-omics datasets. First, we conducted experiments on three labeled cancer multi-omics datasets to verify the SMMSN by assessing the accuracy of the clustering results. Secondly, we tested the performance of the SMMSN on five unlabeled cancer multi-omics datasets through survival analysis and validated the biological significance of the cancer subtypes identified by the SMMSN through multidimensional analysis on two cancer cases.

### 3.1 Multi-omics datasets description

#### 3.1.1 The labeled real-world cancer multi-omics datasets

To demonstrate the effectiveness of SMMSN, we applied it to clustering tasks on three labeled real-world cancer multi-omics datasets. These datasets include the ROSMAP dataset for Alzheimer’s disease (AD) patients and normal control (NC) classification, the Low Grade Glioma (LGG) dataset for Grade 2 and Grade 3 classification in low-grade glioma, and the Pan Kidney Cohort (KIPAN) dataset for the classification of three kidney cancer types: Chromophobe Renal Cell Carcinoma (KICH), Clear Renal Cell Carcinoma (KIRC), and Papillary Renal Cell Carcinoma (KIRP) (Wang et al., 2021). The ROSMAP dataset is composed of ROS and MAP, both of which are longitudinal clinical-pathologic cohort studies of AD from Rush University (Bennett et al., 2012; De Jager et al., 2018). It is available through the AMP-AD Knowledge Portal (https://adknowledgeportal.synapse.org/) (Hodes and Buckholtz, 2016). The omics data for LGG and KIPAN were obtained from TCGA via Broad GDAC Firehose (https://gdac.broadinstitute.org/). For each dataset, we used three types of omics data (i.e., mRNA expression data, DNA methylation data, and miRNA expression data) for clustering to provide comprehensive and complementary information about the diseases. Only samples with matched omics data were included for each data type. Below are detailed descriptions of the datasets.

• ROSMAP: 55889 genes for mRNA expression, 23788 genes for DNA methylation, 309 genes for miRNA expression, 351 patients (NC: 169 patients, AD: 182 patients).
• LGG: 20531 genes for mRNA expression, 20114 genes for DNA methylation, 548 genes for miRNA expression, 510 patients (Grade 2: 246 patients, Grade 3: 264 patients).
• KIPAN: 20531 genes for mRNA expression, 20111 genes for DNA methylation, 445genes for miRNA expression, 658 patients (KICH: 66 patients, KIRC: 318 patients, KIRP: 274 patients).

#### 3.1.2 The unlabeled real-world cancer multi-omics datasets

To further validate the efficacy of SMMSN for cancer subtype discovery, it is used to process multiple omics data sourced from TCGA, as preprocessed by Wang et al. (2014). Our study encompassed five distinct cancer types: Breast Invasive Carcinoma (BIC), Glioblastoma Multiforme (GBM), Lung Squamous Cell Carcinoma (LSCC), Kidney Renal Clear Cell Carcinoma (KRCCC), and Colon Adenocarcinoma (COAD). For each cancer type, we analyzed three types of omics data obtained from different platforms: mRNA expression, DNA methylation, and miRNA expression. Detailed descriptions of these multi-omics datasets for the five cancer types are provided below.

• GBM: 12,042 genes for mRNA expression, 1,305 genes for DNA methylation, 534 genes for miRNA expression, 213 patients.
• BIC: 17,814 genes for mRNA expression, 23,094 genes for DNA methylation, 354 genes for miRNA expression, 105 patients.
• KRCCC: 17,899 genes for mRNA expression, 24,960 genes for DNA methylation, 329 genes for miRNA expression, 122 patients.
• LSCC: 12,042 genes for mRNA expression, 23,074 genes for DNA methylation, 352 genes for miRNA expression, 106 patients.
• COAD: 17,814 genes for mRNA expression, 23,088 genes for DNA methylation, 312 genes for miRNA expression, 92 patients.

### 3.2 Experiment settings

#### 3.2.1 Evaluation indicator

For labeled cancer multi-omics data, we used the Accuracy (ACC) for evaluation to validate the clustering results. ACC quantifies the consistency between the clustering results and the true labels, and its calculation formula is defined as Equation 19:

$$\text{ACC}=\frac{\sum_{i=1}^{n}\delta \left(t_{i},\text{map}\left(y_{i}\right)\right)}{n}$$ (19)

where $t_{i}$ is the true label, $y_{i}$ is the label assigned by the clustering methods, $\delta \left(\cdot \right)$ is the indicator function, which equals 1 when $t_{i}=\text{map}\left(y_{i}\right)$ , and 0 otherwise. $\text{map}\left(\cdot \right)$ represents an optimal mapping function that best matches the clustering labels to the true labels. By calculating ACC, we can intuitively evaluate the clustering performance.

For unlabeled datasets, this study performs survival analysis on cancer subtypes identified through clustering to assess survival disparities among sample groups derived from the proposed algorithm. In statistical analysis, hypothesis testing, such as the Cox Log-rank Test (CLT) (Hosmer et al., 2000), is employed to quantify differences in survival curves. CLT is a non-parametric method commonly used to evaluate whether variations in survival between subtypes are significant. A lower *p*-value from this test suggests stronger evidence against the null hypothesis, indicating substantial differences in survival outcomes that are unlikely to be due to chance alone. Additionally, the Kaplan-Meier estimation method (Hosmer et al., 2000) is utilized to derive survival functions and construct Kaplan-Meier survival curves. These curves plot the survival rate on the *y*-axis against time from the start of observation to the last recorded time point on the *x*-axis. They visually illustrate how the event (e.g., survival or recurrence) unfolds over time for different cancer subtypes, providing insight into their respective prognostic outcomes.

#### 3.2.2 Comparison methods

For comparison purposes, we included five established traditional multi-view clustering algorithms known for their efficacy in cancer subtype prediction: PFA (Shi et al., 2017), SNF (Wang et al., 2014), ANF (Ma and Zhang, 2017), and MVCMO (Yu et al., 2019). Two deep learning-based cancer subtypes discovering methods, Subtype-Former (Yang et al., 2022) and Subtype-DCC (Zhao et al., 2023), are also taken as the competing methods. These algorithms are widely recognized in the field for their ability to integrate diverse data sources and identify meaningful subtypes within cancer datasets.

#### 3.2.3 Experimental parameter settings

The deep learning algorithms involved in this study were implemented using the popular deep learning framework PyTorch 3.9, and all experiments were conducted on an NVIDIA GeForce RTX 4080 GPU with 32 GB RAM, Core I7-12700K. To evaluate the performance of the models and comparison methods, each experiment was run five times, and the average accuracy score along with the standard deviation was reported to ensure the robustness and comparability of the results. The parameter settings for the deep learning models are as follows.

• For the SMMSN algorithm, the network output dimension was set to 100, and the adjustable adjacency matrix parameter *k* was defined as 40. The Adam optimizer was used during training, with an initial learning rate of 1 × 10⁻⁴ and a decay factor of 1 × 10⁻^1^⁵. The model was trained for 500 epochs.
• For the Subtype-DCC algorithm, the feature dimension was set to 256, the batch size to 64, and the number of training epochs to 600. The Adam optimizer was used with automatic learning rate adjustment, starting with an initial learning rate of 1.95 × 10⁻⁴. The instance-level and cluster-level temperature parameters were set to 0.5 and 1.0, respectively.
• For the Subtype-Former algorithm, the Adam optimizer was also used, with an initial learning rate of 7 × 10⁻⁴, a batch size of 8, and the model achieved optimal performance after 45 epochs of training.

For the benchmark machine learning algorithms, they were implemented by MATLAB 2022a software, and their parameters were set strictly according to the guidelines provided by the authors. Each experiment was run five times, and the average accuracy score along with the standard deviation was reported to ensure the robustness and comparability of the results The specific parameters are set as follows.

• For the PFA algorithm, the local sample-spectrum for each biological data type was captured using the *Algorithm_1* function from the *PFA package.* Next, the global sample-spectrum was captured using the *Algorithm_4* function, with the hyperparameter lambda set to 1.
• For the SNF algorithm, an affinity matrix for each omics dataset was calculated using the *dist2* and *affinityMatrix* functions from the *SNFtool* package. The number of neighbors was set to 1/10 of the total number of samples, and the sigma parameter was set to 0.5. These affinity matrices were then integrated using the SNF method with the same number of neighbors and 30 iterations for the multi-omics data. Spectral clustering was performed on the integrated matrix with default parameters.
• For the ANF algorithm, an affinity matrix for each omics dataset was calculated using the *affinity_matrix* function from the *ANFtool* package, with the number of neighbors set to 1/10 of the total number of samples. The matrices were integrated using the ANF method with the same number of neighbors for the multi-omics data.
• For the MVCMO algorithm, an affinity matrix for each omics dataset was calculated using the *knnAffinity* function from the *MVCMO* package, with the number of neighbors set to 5. A fused low-dimensional matrix was then generated using the *adaptedweight* function, with beta set to 1.

#### 3.2.4 Settings of cluster number

For the labeled cancer multi-omics data, the number of clusters corresponds to the number of cancer subtypes in the data itself. The number of clusters for the three datasets, KIPAN, ROSMAP, and LGG, is set to 3, 2, and 2, respectively. For the unlabeled data, we follow the commonly accepted number of cancer subtypes as reported in the majority of studies, such as in references (Wang et al., 2014; Ma and Zhang, 2017; Yu et al., 2019). The number of clusters for the five datasets, GBM, BIC, KRCCC, LSCC, and COAD, is set to 3, 5, 3, 4, and 3, respectively.

### 3.3 Results on labeled multi-omics datasets

Table 1 presents the clustering accuracy of the SMMSN algorithm and competing methods on several labeled cancer multi-omics datasets. In the KIPAN dataset, SMMSN achieved a clustering accuracy of 85.34%, outperforming all other competing methods, especially the two other deep learning models. In comparison, SMMSN’s accuracy was about 3 percentage points higher than the suboptimal method, SNF. This demonstrates SMMSN’s superior ability to distinguish between different types of kidney cancer. In the ROSMAP dataset, SMMSN also exhibited high accuracy, reaching 68.83%, outperforming both classical machine learning models and deep learning models. In the LGG dataset, SMMSN achieved a clustering accuracy of 65.80%. Although DCC (68.39%) performed slightly better than SMMSN, SMMSN still outperformed most of the other methods and maintained stable performance across multiple datasets.

**TABLE 1:** The clustering accuracy (%) of SMMSN and competing methods on several real and labeled cancer multi-omics datasets.

Datasets	Methods			
	SMMSN	PFA	SNF	ANF
KIPAN	**85.34 ± 3.41**	75.81 ± 3.52	82.27 ± 0.00	81.18 ± 0.00
ROSMAP	**68.83 ± 0.71**	61.22 ± 2.98	66.32 ± 0.00	62.64 ± 0.00
LGG	65.80 ± 0.40	60.48 ± 3.85	62.96 ± 0.00	63.41 ± 0.00

Continued	MVSCO	Former	DCC	
KIPAN	79.45 ± 1.55	79.86 ± 0.76	78.66 ± 0.07	
ROSMAP	65.93 ± 2.44	65.01 ± 4.48	64.10 ± 3.80	
LGG	62.32 ± 2.67	64.00 ± 0.40	**68.39 ± 2.55**	

Here Subtype-Former and Subtype-DCC, methods are referred to as Former and DCC, respectively. The best results have been highlighted in bold.

The results indicate that SMMSN consistently outperformed traditional methods such as PFA, SNF, ANF, and MVSCO on all datasets, with particularly noticeable improvements in the KIPAN and ROSMAP datasets. This suggests that SMMSN, by leveraging deep learning’s representation capabilities, better captures the complex nonlinear relationships in multi-omics data and effectively integrates various omics types to improve clustering performance, which is more challenging for traditional algorithms. Compared to other deep learning models, DCC and Former, SMMSN showed significant advantages in the KIPAN and ROSMAP datasets. Although DCC performed slightly better in the LGG dataset, SMMSN demonstrated greater robustness across multiple datasets, with lower standard deviations, indicating more stable performance. SMMSN’s high clustering accuracy highlights its unique advantage in integrating multi-omics data and effectively capturing complementary information between different omics types for cancer subtype classification tasks.

### 3.4 Results on unlabeled multi-omics datasets

#### 3.4.1 Survival analysis on unlabeled multi-omics datasets

**FIGURE 4:**
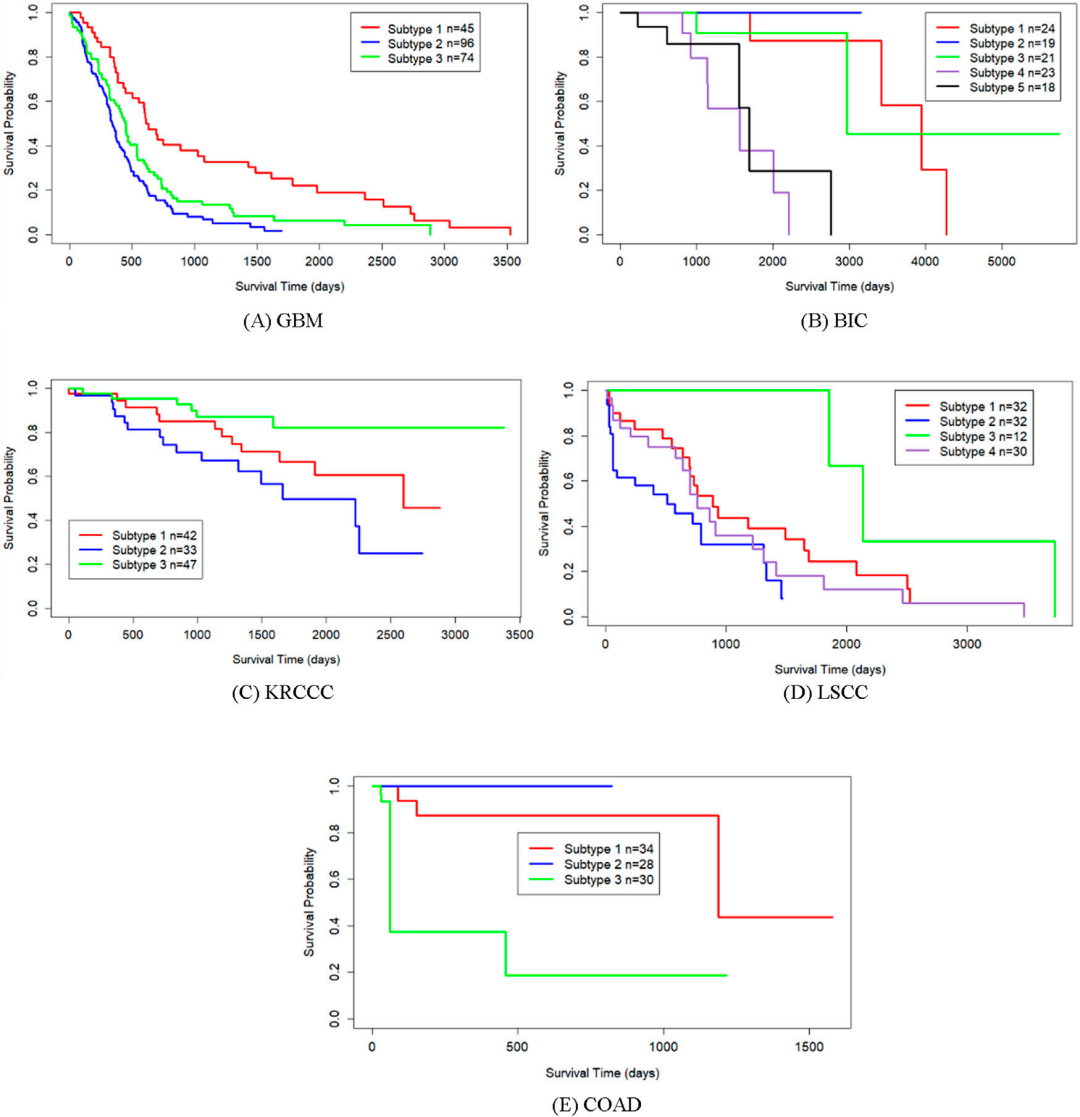
Kaplan-Meier survival curves of discovered subtypes by SMMSN on five datasets. **(A)** GBM **(B)** BIC **(C)** KRCCC **(D)** LSCC **(E)** COAD.

Table 2 shows the *p*-values from survival analysis between SMMSN and competing methods across five datasets. This comparison evaluates the statistical significance of survival differences among cancer subtypes identified by each algorithm. Across all five cancer types, SMMSN consistently yielded the lowest *p*-values compared to other algorithms. Figure 4 presents Kaplan-Meier survival curves generated by SMMSN for each cancer type, depicting the survival trends of respective subtypes. Each curve in Figure 4 illustrates the survival times of distinct cancer subtypes, with sample counts annotated for clarity. These results demonstrate SMMSN’s ability to discern significantly distinct cancer subtypes across various cancer types.

**TABLE 2:** The *p*-values from survival analysis between SMMSN and competing methods across five datasets.

Cancer Types	Methods						
	SMMSN	PFA	SNF	ANF	MVCMO	Former	DCC
GBM	**3.39E-5**	2.15E-4	4.24E-5	4.68E-4	2.14E-3	7.51E-5	2.62E-4
BIC	**7.05E-5**	2.85E-4	7.63E-4	2.65E-4	2.98E-4	1.25E-4	4.63E-4
KRCCC	**6.02E-3**	6.89E-2	3.04E-2	5.17E-2	2.14E-2	1.65E-2	2.67E-2
LSCC	**1.21E-3**	2.04E-2	1.23E-2	9.05E-3	8.97E-3	3.54E-3	2.53E-2
COAD	**5.21E-4**	7.25E-2	3.17E-3	8.78E-3	7.94E-3	2.35E-3	1.58E-3

Here Subtype-Former and Subtype-DCC, methods are referred to as Former and DCC, respectively. The best results have been highlighted in bold.

To further validate the effectiveness of each module in SMMSN, we conducted ablation studies. The SMMSN algorithm mainly consists of three key components: the Feature Representation Fusion (FRF) module based on SAE, the Structural Information Fusion (SIF) module based on GCN, and the Dual Self-supervised (DSS) module. The results of the ablation study are shown in Table 3. It is important to note that when we use only the FRF module or the SIF module, only a single self-supervised learning operation is required, which is denoted as SS module in Table 3. In other words, when both the FRF and SIF modules are used simultaneously in SMMSN, we apply the dual self-supervised module for model learning. From the ablation results shown in Table 3, we can observe different performance outcomes for three different module combinations across five unlabeled multi-omics datasets (GBM, BIC, KRCCC, LSCC, COAD). The analysis can be broken down as follows.

• When only the GCN module and single self-supervised module are used, the results are relatively poor across all datasets, particularly on the KRCCC and LSCC datasets, with p-values of 9.60E-2 and 2.26E-2, respectively. This suggests that while the GCN module can capture structural features, its performance is limited without the feature representation fusion from the SAE module.
• When only the SAE module and single self-supervised module are used, the results are significantly better than the combination with GCN alone, especially on the GBM, KRCCC, and COAD datasets. For example, the error for the KRCCC dataset decreases from 9.60E-2 to 1.79E-2, and for COAD, it reduces from 5.15E-3 to 1.02E-3. This indicates that the feature representation fusion from the SAE module is more effective in capturing the fused characteristics of multi-omics data than structural features alone.
• When SAE, GCN, and the dual self-supervised module are all used together, the errors across all datasets reach their lowest values. For instance, the *p*-value on the KRCCC dataset is further reduced from 1.79E-2 to 6.02E-3, and on LSCC from 9.97E-3 to 1.21E-3, demonstrating that the dual self-supervised module leverages the strengths of both SAE and GCN, greatly enhancing the model’s performance.

**TABLE 3:** Ablation results (*p*-values) of SMMSN on five unlabeled multi-omics datasets.

Components				Cancer types				
SAE	GCN	SS	DSS	GBM	BIC	KRCCC	LSCC	COAD
--	√	√	--	5.77E-4	6.18E-4	9.60E-2	2.26E-2	5.15E-3
√	--	√	--	2.16E-4	2.58E-3	1.79E-2	9.97E-3	1.02E-3
√	√	--	√	3.39E-5	7.05E-5	6.02E-3	1.21E-3	5.21E-4

#### 3.4.2 GBM case analysis

GBM stands as the most prevalent and deadly primary brain tumor in adults, categorized within the glioma group. Numerous studies have extensively explored GBM at the molecular level, identifying distinct cancer subtypes with corresponding clinical implications. For instance, Verhaak et al. (2010) classified GBM based on mRNA expression into four subtypes: Mesenchymal, Classical, Neural, and Proneural. Another study (Noushmehr et al., 2010) differentiated GBM into G-CIMP and non-G-CIMP subtypes based on CpG Island Methylator Phenotype (CIMP).

Using GBM data, we analyzed the distribution of clustering results obtained by SMMSN across the subtypes identified in the aforementioned studies, summarized in Table 4. It is worth noting that the cancer subtypes in references (Verhaak et al., 2010) and (Noushmehr et al., 2010) are classifications derived from different research methods and standards, but they are not considered gold standards for cancer subtypes. Instead, they serve as reference classifications used to help understand and validate the biological differences between the three subtypes identified by the SMMSN algorithm. Table 4 highlights that a majority of patients in subtype 1 align with the Proneural subtype. Subtype 2 shows a closer association with Classical and Proneural subtypes. Subtype 3 predominantly corresponds to the Mesenchymal subtype. It shows that the three subtypes identified have certain differences. Notably, all patients in subtypes 2 and 3 belong to the non-G-CIMP category, while a portion of patients in subtype 1 are classified under G-CIMP. This indicates The difference between the identified subtype 1 and subtype 2–3 (subtype 2 and subtype 3) was obvious, and this conclusion was also verified in Figure 4A that subtype 1 had a longer survival time than subtype 2–3.

**TABLE 4:** The distribution of subtypes identified by SMMSN in relation to those defined in Verhaak et al. (2010) and Noushmehr et al. (2010).

SMMSN subtypes	Subtypes in Verhaak et al. (2010)			Subtypes in Noushmehr et al. (2010)		
	Mesenchymal	Classical	Neural	Proneural	G-CIMP	Non-G-CIMP
Subtype 1	6	9	4	26	19	26
Subtype 2	17	35	16	26	0	94
Subtype 3	42	14	14	4	0	74

The values shown in the table represent the count of patients in each subtype identified by SMMSN, with some association and difference with the classification established by Verhaak et al. (2010) and Noushmehr et al. (2010).

**FIGURE 5:**
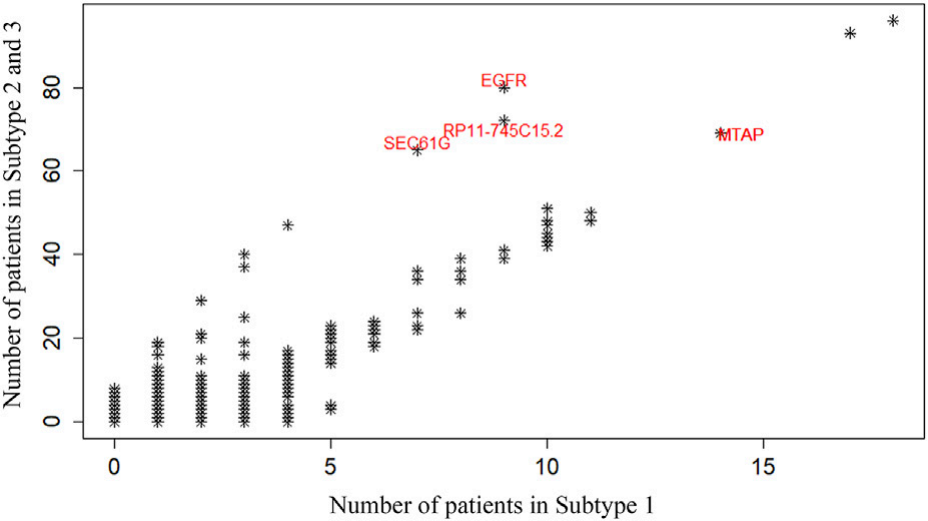
Differences in Copy Number Variation (CNV) abundance of the identified GBM subtypes. Each point represents a gene, and the horizontal and vertical axes show the number of patients carrying a variant of that gene in long-term survival subtype 1 and short-term survival subtype 2–3, respectively.

Subsequently, we further compared long-term survival subtype 1 with short-term survival subtype 2–3 and looked for their differences in gene mutations. Figure 5 show the difference in Copy Number Variation (CNV) abundance between long-lived subtype 1 and short-lived subtype 2–3. In this figure, each point represents a gene, and its axis is the number of patients carrying a variant of that gene in the two survival differential subtypes. The most abundant mutated genes in subtypes 2–3 were EGFR, SEC61G and RP11-745C15.2. EGFR mutations and amplifications are very common in GBM, especially the EGFRvIII variant, which drives rapid tumor cell proliferation, increased invasiveness, and resistance to treatment. EGFR overexpression is closely associated with the progression of more malignant subtypes, which generally indicate poorer survival outcomes (Hu et al., 2022). As a key component of the SEC61 translocation complex in the endoplasmic reticulum, SEC61G is involved in regulating protein transport and processing. Abnormalities in SEC61G may affect proteins involved in cell proliferation and stress responses, thus promoting tumor growth and progression (Zeng et al., 2023). RP11-745C15.2 represents a class of long non-coding RNAs (lncRNAs), whose role in cancer is becoming increasingly recognized. RP11-745C15.2 may regulate key oncogenes like EGFR or its downstream signaling pathways, enhancing malignant cell behavior. In GBM, several lncRNAs, including RP11 family members, are believed to be involved in tumor progression by regulating gene expression, influencing cell growth, and contributing to the aggressive nature of the tumor (Zhang et al., 2020). To sum up, EGFR mutations drive malignant cell proliferation, while SEC61G and RP11-745C15.2, through their roles in protein transport and gene regulation, further promote tumor cell growth, survival, and invasiveness. Their combined action leads to greater gene variation in subtypes 2 and 3, resulting in higher aggressiveness and worse prognosis. This genetic association suggests that these gene alterations are key drivers of tumor progression in the short-term survival subtypes, helping to distinguish subtype 1 (long-term survival) from subtypes 2 and 3 (short-lived).

**FIGURE 6:**
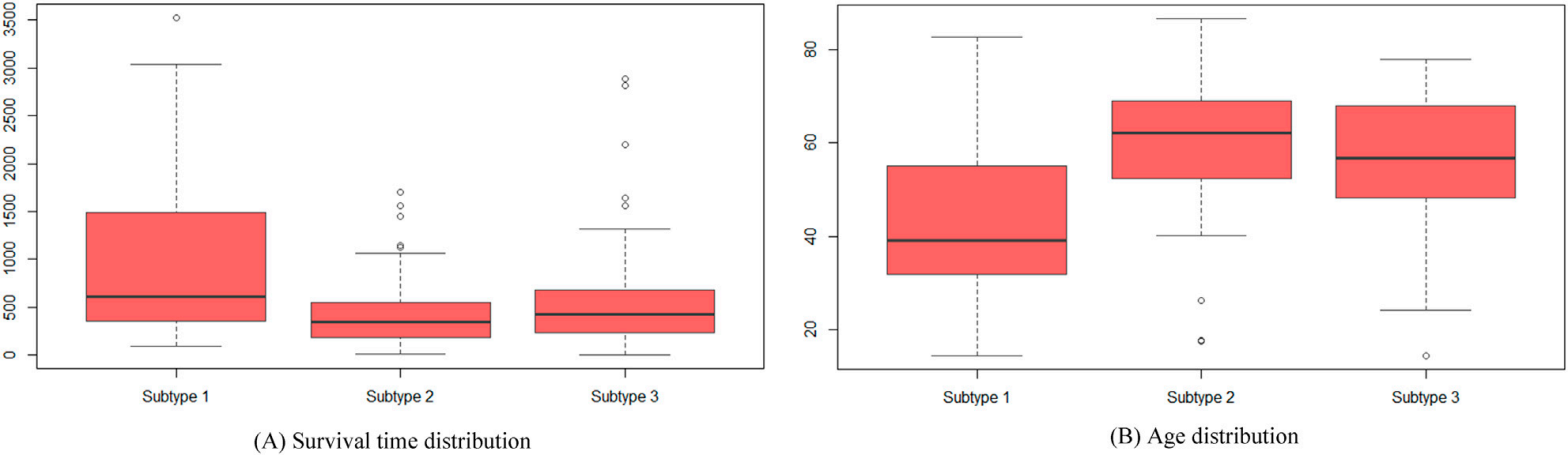
Boxplots used to visualize the distribution of survival time and age among patients classified into the three identified cancer subtypes. **(A)** Displays the variation in survival times across these subtypes, highlighting significant differences. **(B)** Displays the age distributions of patients in each subtype are compared, revealing notable variations among the groups.

Further analysis of the cancer subtypes identified by SMMSN involved accessing clinical data for all GBM patients from the cBio Cancer Genomics Portal database. Figure 6 illustrates boxplots depicting the distribution of survival time and age among these subtypes, demonstrating discernible differences. In Figure 6A, subtype 1 exhibits significantly longer survival compared to subtype 2 and subtype 3, supported by *p*-values from two-sided Welch’s t-tests: 1.45E-4 and 5.69E-3, respectively. Figure 6B reveals that patients in subtype 1 are younger than those in subtype 2 and subtype 3, with corresponding *t*-test *p*-values of 2.37E-7 and 5.21E-5, respectively. Moreover, we conducted an Analysis of Variance (ANOVA) test across the three subtypes, confirming significant differences in both survival time (*p* = 8.24E-7) and age distribution (*p* = 2.48E-9). Similarly, the Kruskal–Wallis test also indicated statistically significant distinctions in age (*p* = 1.97E-7) and survival time (*p* = 1.84E-04) among the subtypes. These consistent findings underscore the biological relevance and statistical significance of the identified subtypes with respect to both age demographics and survival outcomes.

**FIGURE 7:**
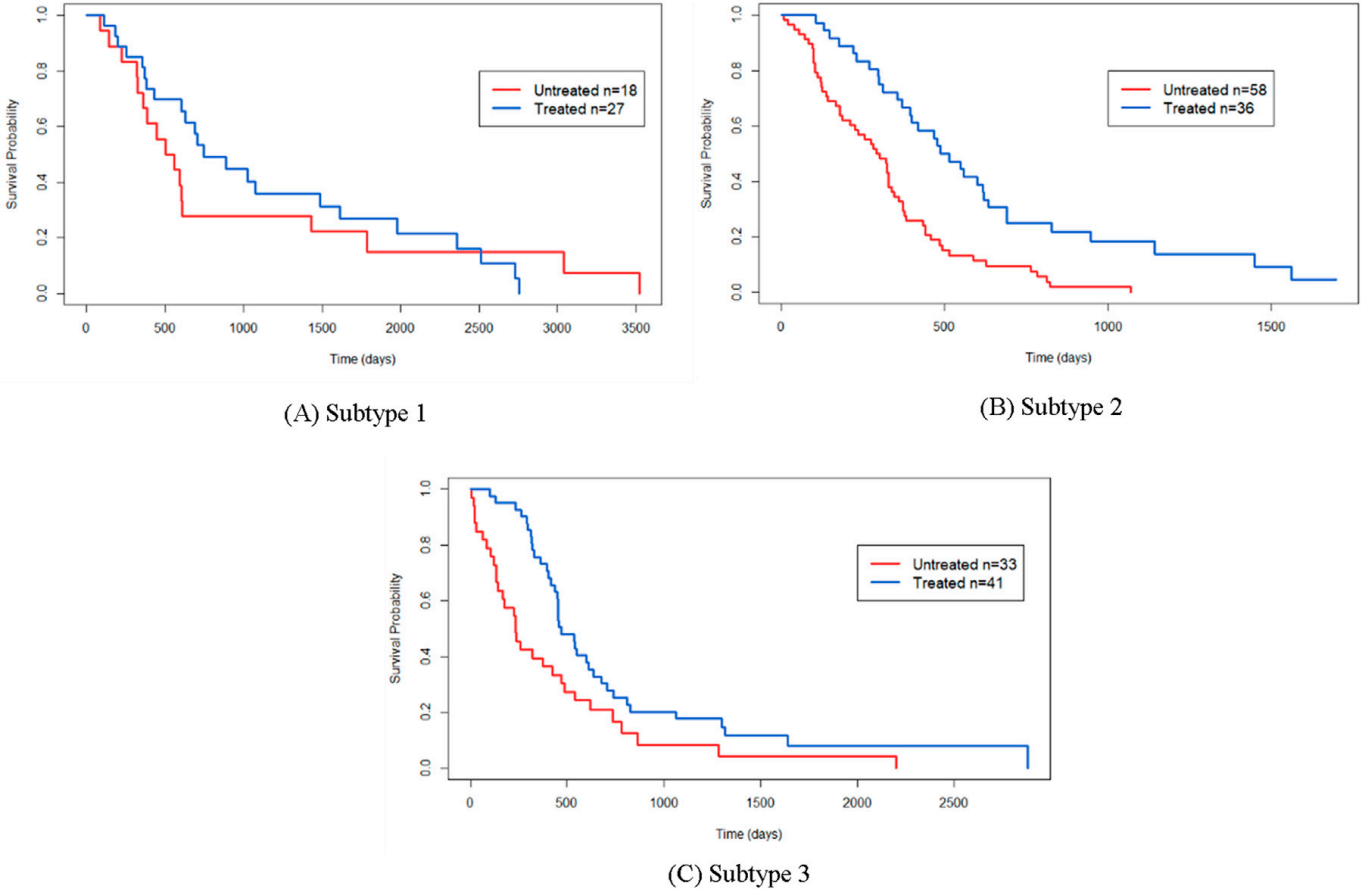
Here are the Kaplan-Meier survival curves depicting the response to Temozolomide (TMZ) for the identified cancer subtypes by SMMSN: **(A)** Kaplan-Meier survival curve for Sub-type 1 in response to TMZ. **(B)** Kaplan-Meier survival curve for Subtype 2 in response to TMZ. **(C)** Kaplan-Meier survival curve for Subtype 3 in response to TMZ.

Figure 7 presents Kaplan-Meier survival curves depicting the response of patients to the drug Temozolomide (TMZ). Patients are stratified into two groups: those treated with TMZ and those not treated with TMZ. The *p*-values for subtype 1, subtype 2, and subtype 3 are 0.65, 4.12E-5, and 5.42E-2, respectively. These results indicate that TMZ treatment has minimal impact on the survival outcomes of patients in subtype 1, while it significantly affects the survival of patients in subtypes 2 and 3.

**FIGURE 8:**
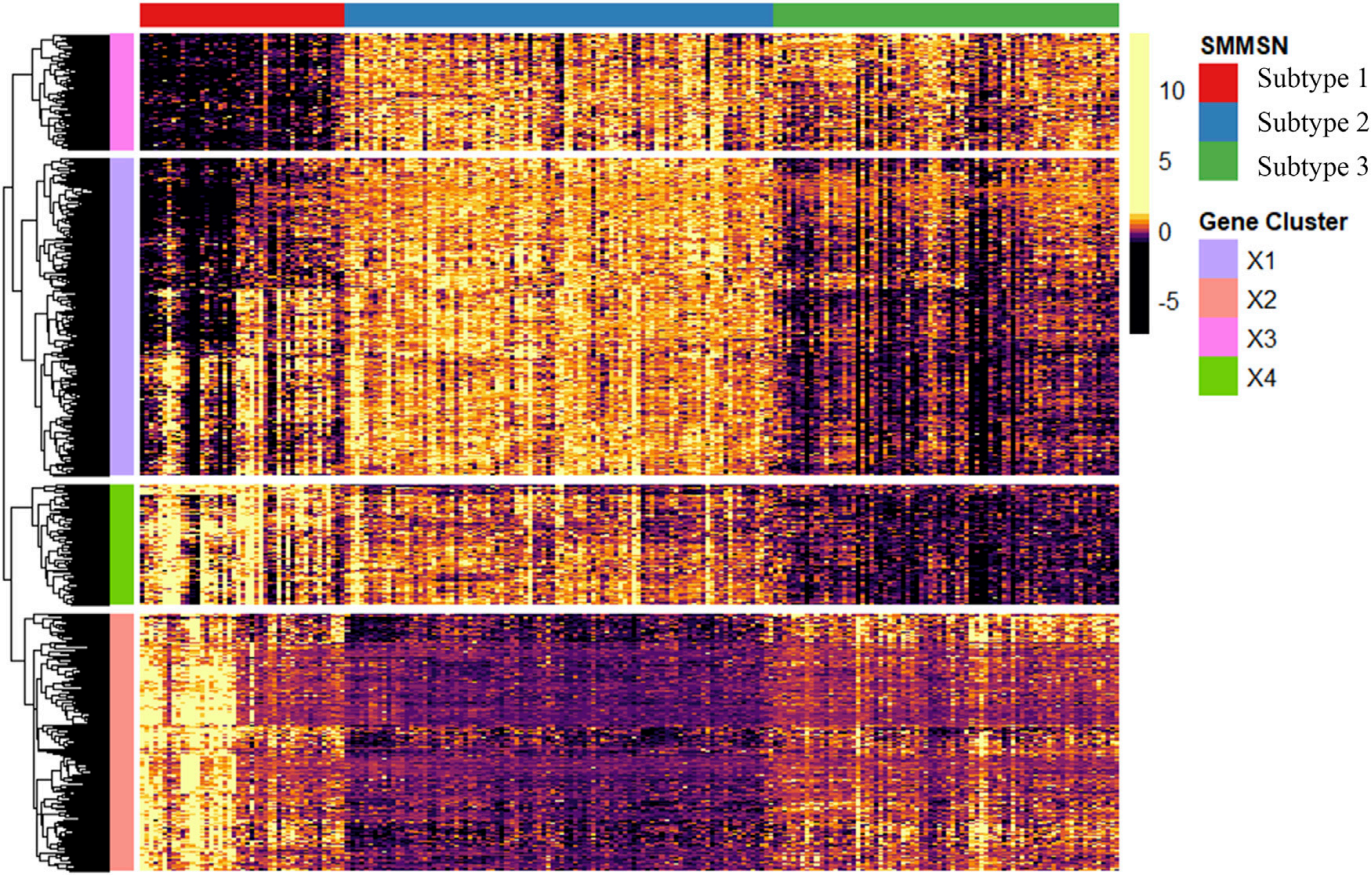
The heatmap showcases the top 1,000 genes whose mRNA expression varies significantly across the three subtypes identified by SMMSN in GBM. Subtype 1, Subtype 2, and Subtype 3 are represented by different colors respectively, highlighting distinct clusters of gene expression patterns among the subtypes.

Differential gene expression and GO enrichment analyses were conducted on GBM data to assess differences among the three subtypes identified by SMMSN. Initially, significant differentially expressed genes across the subtypes were identified using the ANOVA method. Figure 8 displays a heatmap illustrating the top 1,000 differentially expressed genes in mRNA expression data, with panels A, B, and C representing subtype 1, subtype 2, and subtype 3, respectively. The heatmap reveals distinct clusters among the differentially expressed genes, indicating subtype-specific expression patterns.

**FIGURE 9:**
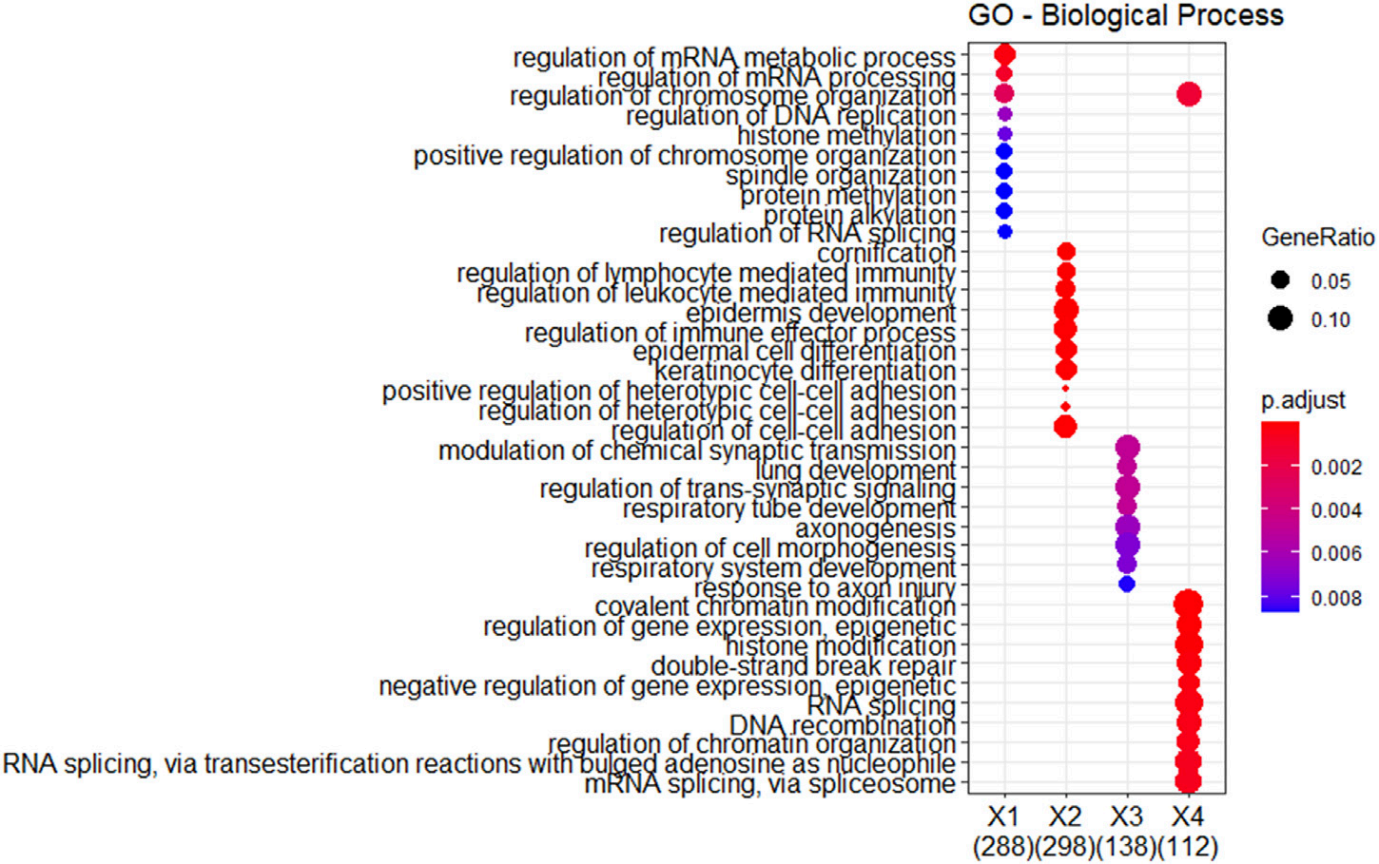
Functional enrichment analysis was performed on the differentially expressed genes identified in Figure 8.

Further analysis involved functional enrichment of these differentially expressed genes. Figure 9 presents the results of GO enrichment analysis categorizing the genes into four distinct groups (X1, X2, X3, and X4). Each group is associated with specific GO biological processes, as indicated by the number of enriched genes listed below. Notably, genes related to the regulation of mRNA metabolism/chromosome organization were downregulated in subtype 3 and upregulated in subtype 2. The genes related to the regulation of immune effector process/leukocyte-mediated immunity/lymphocyte-mediated immunity and hetero-cell bonding were downregulated in subtype 2. Genes associated with DNA recombination, nuclear transport/export, and RNA splicing were downregulated in subtype 3. In conclusion, GBM subtypes identified by SMMSN have obvious differences in clinical indicators and molecular levels.

#### 3.4.3 BIC case analysis

BIC refers to a malignancy in which cancer cells have penetrated the basement membrane of breast ducts or lobular acinus and invaded the stroma. Similar to the GBM case analysis procedure described above, we first made a comparison with previous BIC subtype study. PAM50 is a molecular subtype of BIC based on quantitative detection of the expression levels of 50 functional genes in breast tumor tissues, including Luminal A, Luminal B, HER2-enriched and Basal-like subtypes (Parker et al., 2009). Table 5 depicts the comparison results of the distribution of BIC subtypes identified by SMMSN in PAM50 subtypes.

**TABLE 5:** The distribution of subtypes identified by SMMSN in PAM50 subtypes.

SMMSN subtypes	Subtypes in Parker et al. (2009)			
	Luminal A	Luminal B	Basal-like	HER2-enriched
Subtype 1	11	2	6	4
Subtype 2	14	3	0	0
Subtype 3	13	0	1	4
Subtype 4	13	7	0	3
Subtype 5	2	0	16	0

From Table 5, it can be observed that the five cancer subtypes identified by the SMMSN algorithm show different distribution patterns in the PAM50 subtypes (Luminal A, Luminal B, Basal-like, HER2-enriched) from the study in (Parker et al., 2009). SMMSN subtypes 1, 2, 3, and 4 are mainly concentrated in Luminal A, with subtype 2 almost entirely composed of Luminal A patients, indicating a high level of consistency between these subtypes and the Luminal A subtype. SMMSN subtype 5, on the other hand, is predominantly composed of Basal-like patients, suggesting a strong correspondence with the Basal-like subtype. In contrast, Luminal B and HER2-enriched patients are more dispersed across multiple SMMSN subtypes, especially in subtypes 1 and 4, revealing a certain degree of discrepancy between the subtypes identified by SMMSN and the PAM50 subtypes. These observations reflect a strong alignment between SMMSN subtypes and PAM50 in certain subtypes, while in others, cross-subtype distribution patterns are apparent.

**FIGURE 10:**
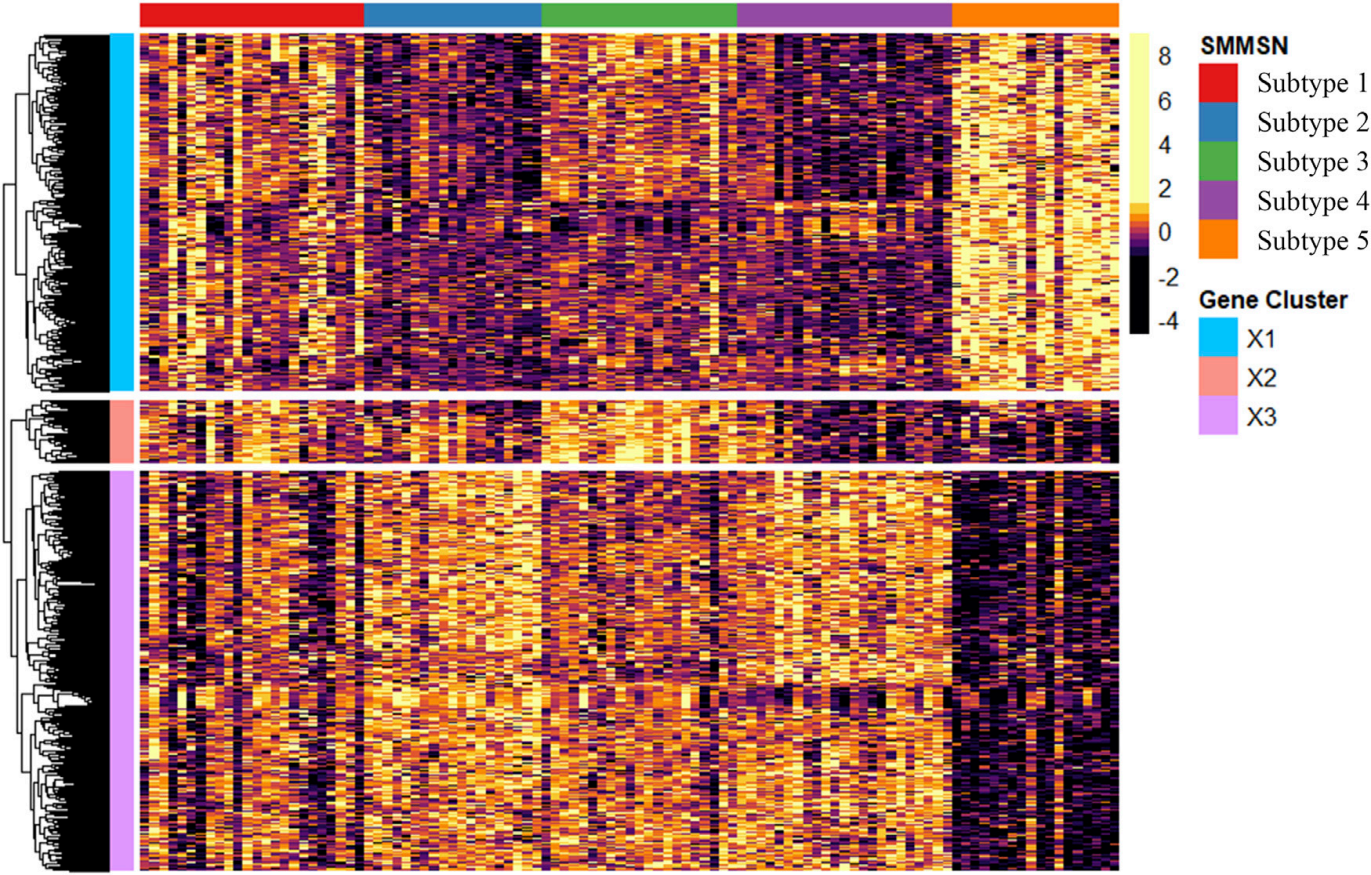
The heatmap showcases the top 1,000 genes whose mRNA expression varies significantly across the three subtypes identified by SMMSN in BIC.

**FIGURE 11:**
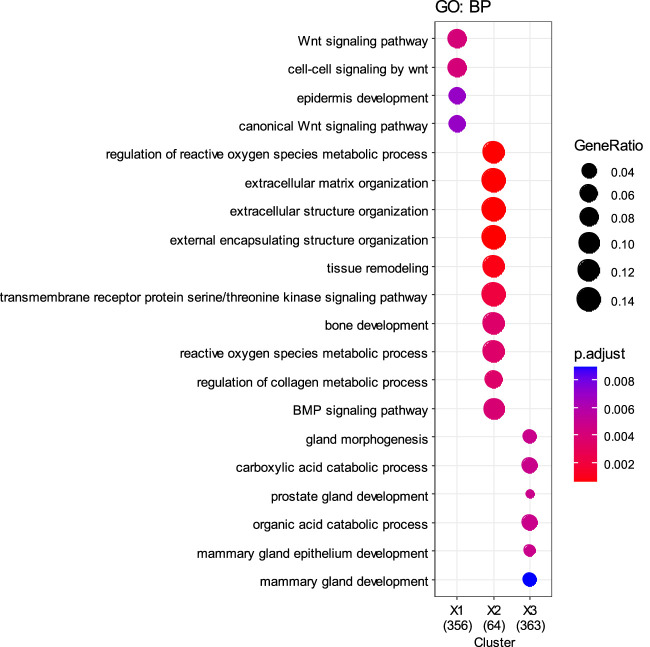
Functional enrichment analysis was performed on the differentially expressed genes identified in Figure 10.

To further validate the biological differences among the cancer subtypes 1, 2, 3, 4, and 5 identified by SMMSN, especially the first four subtypes, differential expression analysis of BIC expression data was performed using the Kruskal–Wallis test. Hierarchical clustering method was used to group the top 1,000 differential genes of BIC, and GO: BP analysis was performed based on the grouping results, and their results are presented in Figures 10, 11. Figure 10 presents the heatmap of the top 1,000 genes whose mRNA expression varies significantly across the five subtypes identified by SMMSN in BIC. It can be observed that there exists distinct differences in gene expression among the five subtypes. Figure 11 shows the functional enrichment analysis on the differentially expressed genes identified in Figure 10. It can be found that genes related to Wnt signaling pathway and epidermal development are upregulated in subtype 5 and downregulated in subtype 2 and 4. Genes related to extracellular matrix/structural organization, metabolic processes, tissue remodeling and bone development were downregulated in subtypes 4 and 5. Genes related to prostate/breast development and organic/carboxylic acid catabolic processes were downregulated in subtype 5. The differential expression analysis reveals significant gene expression differences among the five cancer subtypes identified by SMMSN. Functional enrichment analysis shows distinct up- and downregulation patterns across the subtypes, highlighting their biological divergence.

## 4 Conclusion

Over the past decades, numerous models integrating multi-view biological data, utilizing technologies have been developed and applied to various bioinformatics challenges. These studies have provided valuable insights into understanding the etiology and progression of cancer. Effective mining of cancer subtypes based on biological characteristics from multi-omics data is crucial in bioinformatics research.

In this paper, we introduce a novel method for predicting cancer subtypes called Self-supervised Multi-fusion Strategy Network (SMMSN). SMMSN leverages Stacked Autoencoder (SAE) and Graph Convolutional Network (GCN) modules to learn high-level feature representations and structural representations from each omics data type, respectively. These representations are then integrated to capture comprehensive information across different omics data using two fusion methods: error reconstruction and adaptive weighting network. A dual self-supervised module is employed to jointly train SAE and GCN in an end-to-end manner. Upon convergence, the SMMSN model yields clustering results. We validate the efficacy of SMMSN using 8 real-world cancer datasets, including both labeled and unlabeled multi-omics data, demonstrating its superior performance compared to existing integration methods. Specifically, on GBM data and BIC data, extensive studies confirm that the cancer subtypes predicted by SMMSN exhibit significant and biologically meaningful differences. This underscores the capability of SMMSN to effectively integrate multi-omics data and enhance the understanding of cancer heterogeneity and subtype classification.

Our future research directions could focus on enhancing the interpretability and robustness of the SMMSN model, exploring its application across additional cancer types and expanding its utility in personalized medicine through integration with clinical data.

## Data Availability

The original contributions presented in the study are included in the article/supplementary material, further inquiries can be directed to the corresponding author.

## References

[B1] Akbani R. Ng K. S. Werner H. M. Fan Z. Mills G. B. Liu W. (2014). Abstract 4262: a pan-cancer proteomic analysis of the Cancer Genome Atlas (TCGA) project. Cancer Res. 74, 4262. 10.1158/1538-7445.am2014-4262

[B2] Baird T. Roychoudhuri R. (2024). GS-TCGA: gene set-based analysis of the cancer genome atlas. J. Comput. Biol. 31 (3), 229–240. 10.1089/cmb.2023.0278 38436570

[B3] Bennett D. A. Schneider J. A. Arvanitakis Z. Wilson R. S. (2012). Overview and findings from the religious orders study. Curr. Alzheimer Res. 9 (6), 628–645. 10.2174/156720512801322573 22471860 PMC3409291

[B4] Bruna J. Zaremba W. Szlam A. LeCun Y. (2013). Spectral networks and locally connected networks on graphs. arXiv Prepr. arXiv:1312.6203. 10.48550/arXiv.1312.6203

[B5] Chen J. Zhu J. Song L. (2017). Stochastic training of graph convolutional networks with variance reduction. arXiv Prepr. arXiv:1710.10568. 10.48550/arXiv.1710.10568

[B6] Chen R. Yang L. Goodison S. Sun Y. (2020). Deep-learning approach to identifying cancer subtypes using high-dimensional genomic data. Bioinformatics 35, 1476–1483. 10.1093/bioinformatics/btz769 PMC8215925 31603461

[B7] Chen Y. Liu M. Wang Y. (2023). Bioinformatic analysis reveals lysosome-related biomarkers and molecular subtypes in preeclampsia: novel insights into the pathogenesis of preeclampsia. Front. Genet. 14, 1228110. 10.3389/fgene.2023.1228110 37576559 PMC10416227

[B8] Dai H. Kozareva Z. Dai B. Smola A. Song L. (2018). “PMLR. Learning steady-states of iterative algorithms over graphs,” in In International Conference on Machine Learning, 1106–1114.

[B9] Defferrard M. Bresson X. Vandergheynst P. (2016). Convolutional neural networks on graphs with fast localized spectral filtering. Proc. 30th Int. Conf. Neural Inf. Process. Syst. 29, 3844–3852. 10.48550/arXiv.1606.09375

[B10] De Jager P. L. Ma Y. McCabe C. Xu J. Vardarajan B. N. Felsky D. (2018). A multi-omic atlas of the human frontal cortex for aging and Alzheimer’s disease research. Sci. Data 5 (1), 180142–180213. 10.1038/sdata.2018.142 30084846 PMC6080491

[B11] Dunnett C. W. Sobel M. (1954). A bivariate generalization of Student's t-distribution, with tables for certain special cases. Biometrika 41, 153–169. 10.2307/2333013

[B12] Grover A. Leskovec J. (2016). “Node2vec: scalable feature learning for networks,” in Proceedings of the 20th ACM International Conference on Knowledge Discovery and Data Mining, 855–864. 10.1145/2939672.2939754 PMC5108654 27853626

[B13] Hamilton W. L. Ying Z. Leskovec J. (2017). Inductive representation learning on large graphs. Adv. Neural Inf. Process. Syst. 30, 1024–1034. 10.48550/arXiv.1706.02216

[B14] Hodes R. J. Buckholtz N. (2016). Accelerating medicines partnership: Alzheimer’s disease (AMP-AD) knowledge portal aids Alzheimer’s drug discovery through open data sharing. Expert Opin. Ther. Targets 20 (4), 389–391. 10.1517/14728222.2016.1135132 26853544

[B15] Hosmer D. W. Lemeshow S. May S. (2000). Applied survival analysis: regression modeling of time to event data. J. Stat. Plan. Inference 91, 173–175. 10.1016/s0378-3758(00)00130-0

[B16] Hu C. Leche C. A. Kiyatkin A. Yu Z. Stayrook S. E. Ferguson K. M. (2022). Glioblastoma mutations alter EGFR dimer structure to prevent ligand bias. Nature 602 (7897), 518–522. 10.1038/s41586-021-04393-3 35140400 PMC8857055

[B17] Jin H. Wang L. Bernards R. (2023). Rational combinations of targeted cancer therapies: background, advances and challenges. Nat. Rev. Drug Discov. 22 (3), 213–234. 10.1038/s41573-022-00615-z 36509911

[B18] Livesey M. Eshibona N. Bendou H. (2023). Assessment of the progression of kidney renal clear cell carcinoma using transcriptional profiles revealed new cancer subtypes with variable prognosis. Front. Genet. 14, 1291043. 10.3389/fgene.2023.1291043 38075696 PMC10704507

[B19] Luxburg U. (2007). A tutorial on spectral clustering. Statistics Comput. 17, 395–416. 10.1007/s11222-007-9033-z

[B20] Ma T. Zhang A. (2017). “Integrate multi-omic data using affinity network fusion (ANF) for cancer patient clustering,” in 2017 IEEE International Conference on Bioinformatics and Biomedicine (BIBM), 398–403. 10.1109/bibm.2017.8217682

[B21] Mo Q. Wang S. Seshan V. E. Olshen A. B. Schultz N. Sander C. (2013). Pattern discovery and cancer gene identification in integrated cancer genomic data. Proc. Natl. Acad. Sci. U. S. A. 110, 4245–4250. 10.1073/pnas.1208949110 23431203 PMC3600490

[B22] Noushmehr H. Weisenberger D. J. Diefes K. Phillips H. S. Pujara K. Berman B. P. (2010). Identification of a CpG island methylator phenotype that defines a distinct subgroup of glioma. Cancer Cell 17, 510–522. 10.1016/j.ccr.2010.03.017 20399149 PMC2872684

[B23] Parker J. S. Mullins M. Cheang M. C. Leung S. Voduc D. Vickery T. (2009). Supervised risk predictor of breast cancer based on intrinsic subtypes. J. Clin. Oncol. 27 (8), 1160–1167. 10.1200/JCO.2008.18.1370 19204204 PMC2667820

[B24] Perozzi B. Al-Rfou R. Skiena S. (2014). “Deepwalk: online learning of social representations,” in Proceedings of the 20th ACM International Conference on Knowledge Discovery and Data Mining, 701–710. 10.1145/2623330.2623732

[B25] Rappoport N. Shamir R. (2018). Multi-omic and multi-view clustering algorithms: review and cancer benchmark. Nucleic Acids Res. 46, 10546–10562. 10.1093/nar/gky889 30295871 PMC6237755

[B26] Shen R. Olshen A. B. Ladanyi M. (2009). Integrative clustering of multiple genomic data types using a joint latent variable model with application to breast and lung cancer subtype analysis. Bioinformatics 25, 2906–2912. 10.1093/bioinformatics/btp543 19759197 PMC2800366

[B27] Shi Q. Zhang C. Peng M. Yu X. Zeng T. Liu J. (2017). Pattern fusion analysis by adaptive alignment of multiple heterogeneous omics data. Bioinformatics 33, 2706–2714. 10.1093/bioinformatics/btx176 28520848

[B28] Sosinsky A. Ambrose J. Cross W. Turnbull C. Henderson S. Jones L. (2024). Insights for precision oncology from the integration of genomic and clinical data of 13,880 tumors from the 100,000 Genomes Cancer Programme. Nat. Med. 30 (1), 279–289. 10.1038/s41591-023-02682-0 38200255 PMC10803271

[B29] Tang J. Qu M. Wang M. Zhang M. Yan J. Mei Q. (2015). “Line: large-scale information network embedding,” in Proceedings of the 24th International Conference on World Wide Web, 1067–1077. 10.1145/2736277.2741093

[B30] Tao Z. Liu H. Li J. Wang Z. Fu Y. (2019). “Adversarial graph embedding for ensemble clustering,” in Proceedings of the Twenty-Eighth International Joint Conference on Artificial Intelligence, 3562–3568. 10.24963/ijcai.2019/494

[B31] Thomas N. Kipf M. W. (2017). “Semi-supervised classification with graph convolutional networks,” in Proceedings of International Conference on Learning Representations, 1–14.

[B32] Veličković P. Cucurull G. Casanova A. Romero A. Lio P. Bengio Y. (2017). Graph attention networks. arXiv Prepr. arXiv:1710.10903. 10.48550/arXiv.1710.10903

[B33] Verhaak R. G. W. Hoadley K. A. Purdom E. Wang V. Qi Y. Wilkerson M. D. (2010). Integrated genomic analysis identifies clinically relevant subtypes of glioblastoma characterized by abnormalities in PDGFRA, IDH1, EGFR, and NF1. Cancer Cell 17, 98–110. 10.1016/j.ccr.2009.12.020 20129251 PMC2818769

[B34] Wang B. Mezlini A. M. Demir F. Fiume M. Tu Z. Brudno M. (2014). Similarity network fusion for aggregating data types on a genomic scale. Nat. Methods 11, 333–337. 10.1038/nmeth.2810 24464287

[B35] Wang D. Liu B. Zhang Z. (2023). Accelerating the understanding of cancer biology through the lens of genomics. Cell 186 (8), 1755–1771. 10.1016/j.cell.2023.02.015 37059071

[B36] Wang Q. Ding Z. Tao Z. Gao Q. Yun F. (2018). “Partial multi-view clustering via consistent GAN,” in Proceedings of the 2018 IEEE International Conference on Data Mining (ICDM), 1290–1295.

[B37] Wang T. Shao W. Huang Z. Tang H. Zhang J. Ding Z. (2021). MOGONET integrates multi-omics data using graph convolutional networks allowing patient classification and biomarker identification. Nat. Commun. 12 (1), 3445. 10.1038/s41467-021-23774-w 34103512 PMC8187432

[B38] Way G. P. Greene C. S. (2018). Extracting a biologically relevant latent space from cancer transcriptomes with variational autoencoders. Pac Symp. Biocomput 23, 80–91. 10.1142/9789813235533_0008 29218871 PMC5728678

[B39] Wu D. Wang D. Zhang M. Q. Gu J. (2015). Fast dimension reduction and integrative clustering of multi-omics data using low-rank approximation: application to cancer molecular classification. BMC Genomics 16, 1022. 10.1186/s12864-015-2223-8 26626453 PMC4667498

[B40] Xu J. Wu P. Chen Y. Meng Q. Dawood H. (2019). A hierarchical integration deep flexible neural forest framework for cancer subtype classification by integrating multi-omics data. BMC Bioinforma. 20, 527. 10.1186/s12859-019-3116-7 PMC6819613 31660856

[B41] Xu X. Peng Q. Jiang X. Tan S. Yang Y. Yang W. (2023). Metabolic reprogramming and epigenetic modifications in cancer: from the impacts and mechanisms to the treatment potential. Exp. and Mol. Med. 55 (7), 1357–1370. 10.1038/s12276-023-01020-1 37394582 PMC10394076

[B42] Yang H. Sheng Y. Jiang Y. Fang X. Li D. Zhang J. (2022). Subtype-former: a deep learning approach for cancer subtype discovery with multi-omics data. arxiv Prepr. arxiv:2207.14639. 10.48550/arXiv.2207.14639

[B43] Yu Y. Zhang L. H. Zhang S. (2019). Simultaneous clustering of multiview biomedical data using manifold optimization. Bioinformatics 35, 4029–4037. 10.1093/bioinformatics/btz217 30918942

[B44] Zeng K. Zeng Y. Zhan H. Zhan Z. Wang L. Tang Y. (2023). SEC61G assists EGFR-amplified glioblastoma to evade immune elimination. Proc. Natl. Acad. Sci. 120 (32), e2303400120. 10.1073/pnas.2303400120 37523556 PMC10410745

[B45] Zhang Y. Mou C. Shang M. Jiang M. Xu C. (2020). Long noncoding RNA RP11‐626G11. 3 promotes the progression of glioma through miR‐375‐SP1 axis. Mol. Carcinog. 59 (5), 492–502. 10.1002/mc.23173 32128886

[B46] Zhao J. Zhao B. Song X. Lyu C. Chen W. Xiong Y. (2023). Subtype-DCC: decoupled contrastive clustering method for cancer subtype identification based on multi-omics data. Briefings Bioinforma. 24 (2), bbad025. 10.1093/bib/bbad025 36702755

